# A novel non sense mutation in *WDR62* causes autosomal recessive primary microcephaly: a case report

**DOI:** 10.1186/s12881-018-0625-6

**Published:** 2018-07-18

**Authors:** Imane Cherkaoui Jaouad, Abdelali Zrhidri, Wafaa Jdioui, Jaber Lyahyai, Laure Raymond, Grégory Egéa, Mohamed Taoudi, Said El Mouatassim, Abdelaziz Sefiani

**Affiliations:** 1 Centre de Génomique Humaine, Faculté de Médecine et de Pharmacie, Université Mohammed V, Rabat, Morocco; 2 Département de Génétique Médicale, Institut National d’Hygiène, Rabat, Morocco; 3 Département de Génétique Moléculaire, Laboratoire Biomnis, Lyon, France

**Keywords:** Autosomal recessive primary microcephaly, Genetic heterogeneity, *WDR62*, Whole exome sequencing

## Abstract

**Background:**

Autosomal recessive primary microcephaly (MCPH) is a rare genetically heterogeneous disorder of neurogenic brain development characterized by a reduced head circumference at birth with no remarkable anomalies of brain architecture and variable degrees of intellectual impairment. Clinical and genetic heterogeneity in genetic disorders represent a major diagnostic challenge.

**Case presentation:**

Two patients, 11 and 9 years old, born from consanguineous parents, were referred to the department of medical genetics at the National Institute of Health in Rabat. The diagnosis of MCPH was made, based on reduced head circumference without brain architecture abnormalities. The two patients were subject to the whole-exome sequencing, which allowed to diagnose a novel homozygous mutation c.1027C > T; p.Gln343* in exon 8 of *WDR62*, a gene already known to be related to MCPH. Sanger sequencing confirmed the segregation of the mutation in the family.

**Conclusion:**

Our data expends the spectrum of mutations in *WDR62* gene, proves the efficiency and cost-effectiveness of whole exome sequencing for the molecular diagnosis of genetically heterogeneous disorders such MCPH. Exome sequencing led to the rapid and cost-effective identification of a novel homozygous mutation in *WDR62* gene, thereby facilitating genetic counseling.

## Background

Autosomal recessive primary microcephaly (MCPH) [1], also known as Microcephaly vera, is a rare genetically heterogeneous disorder of neurogenic brain development characterized by a reduced head circumference at birth equal to or less than − 2 standard deviation (SD) below the mean for sex, age and ethnicity and results from intrauterine reduced brain growth. It is associated with non progressive mental retardation of variable degree, minimal neurological deficit, limited to a mild pyramidal syndrome such as mild spasticity of the lower limbs, without additional severe brain and visceral malformations.

The incidence of MCPH is relatively high, approximately 1 per 10,000, especially in regions with high rate of consanguinity, such as Morocco [2, 3].

MCPH exhibits genetic heterogeneity, and to date, 16 genes underlying MCPH have been identified with mutations in the *ASPM* gene accounting for 50% of cases [4–6], followed in frequency by mutations in *WDR62* [7, 8].

Genetic heterogeneity in MCPH represents a major diagnostic challenge. Here, we used the whole exome sequencing (WES) as a diagnostic approach for establishing a molecular diagnosis in a consanguineous Moroccan family with two MCPH affected children.

## Case presentation

Two individuals of a consanguineous family from the south of Morocco, displayed phenotypical and behavioral characteristics of primary microcephaly, especially, a reduction of head circumference, intellectual disability, speech delay, aggressiveness and hyperactive behavior.

An autosomal recessive inheritance was expected according to the pedigree.

**Fig. 1:**
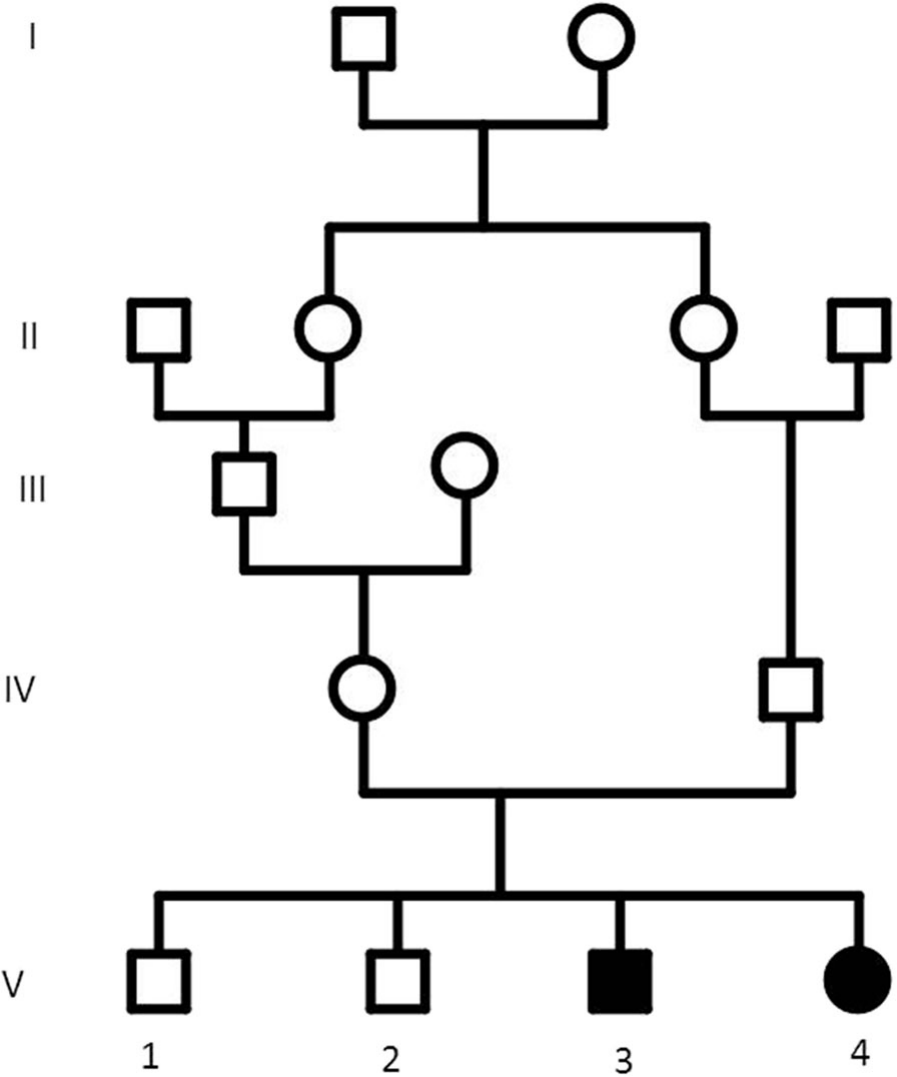
Pedigree of the studied family. Filled symbols represent affected individuals and open symbols represent unaffected individuals

Patient V.3 is a 11 years old boy (Patient V.3, Fig. 1), was born at full term by a normal vaginal delivery after uneventful pregnancy, antenatal ultra-sound revealed microcephaly and postnatal head circumference was 30 cm (< 5th centile) and birth weight: 2.8 kg (< 10th centile). He did not suffer any significant postnatal problem. For the developmental history; the patient walked independently at 3 years of age and suffered considerable delayed fine motor skills, mostly a difficulty in the hand-eye coordination movements like writing, zipping a zipper or folding clothes. At clinical examination at the age of 11, this patient presented with short stature at 120 cm (< 5th centile), weight delay at 30 kg (< 5th centile), and head circumference at 45.5 cm (< 5th centile).

Patient V.4 is a female (Patient V.4, Fig. 1), 9 years old, who had a similar clinical presentation to her brother (patient V.3); showing developmental delay and microcephaly. She had a reduced head circumference of 42 cm (< 5th centile) and a height of 110 cm (< 5th centile), with aggressiveness and excess salivary production. She was born at term, by normal vaginal delivery after normal pregnancy with birth weight: 2.750 kg and head circumference: 29.5 cm.

Otherwise, the neurological history in the two siblings didn’t reveal any symptom of hypotonia, seizures, ataxia or cerebral palsy. Moreover, the ocular checking with fundus examination for the two patients proved normal.

Magnetic resonance imaging scan of the two patients showed a reduced volume of the two cerebral hemispheres with no brain architecture abnormalities, suggesting a proportionately small-sized brain. Based on clinical information and pedigree (Fig. 1) the patients were diagnosed with primary autosomal recessive microcephaly.

Informed consent was obtained from the parents prior to initiation of laboratory work. Peripheral blood was collected from the probands and their parents. Genomic DNA was extracted from blood using QIAamp DNA Blood Mini Kit (Qiagen Valencia, CA). WES was performed in probands (Patients V.3 and V.4, Fig. 1); 500 ng of fragmented DNA (enzymatic fragmentation, Kapa Hyper Plus Kit) was amplified in compliance with user guide, and was subjected to enrichment with SeqCap EZ Human Exome v3.0 (Roche Nimblegen). The 64 enriched megabases were sequenced using an Illumina HiSeq 2500 system in rapid run paired-end mode (2x100bp). Raw data (bcl files) was converted to FASTQ files using bcl2fastq v1.8.4 (Illumina). Sequences were analyzed as recommended by GATK best practices: mapping was performed using BWA-MEM, variant calling using GATK (haplotype caller). Annotation and filtering steps were performed using VariantStudio (Illumina). Variants files of parents and index case were confronted: only variants that fulfilled recessive inheritance pattern were selected. Among them, variants with allele frequencies above 1% in ESP6500 exome project, and variants not predicted to be deleterious were excluded.

The established variants were cross-checked with the 1000 genomes database (http://www.1000genomes.org/), with the Exome Variant Server (http://evs.gs.washington.edu/EVS/), HGMD (http://www.biobase-international.com/product/hgmd) and with the « clinvar » database (http://www.ncbi.nlm.nih.gov/clinvar/).

**Fig. 2:**
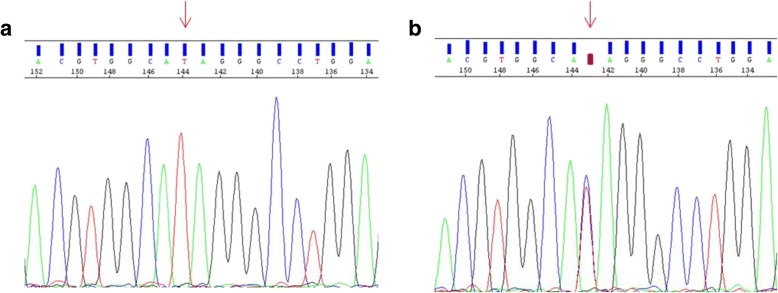
Electrophoregrams showing the c.1027C > T homozygous mutation in the patient (**a**) and the heterozygous mutation in the parents (**b**). Arrows indicate the region of the mutation

Upon WES, we detected a homozygous mutation c.1027C > T; p.Gln343* in exon 8 of the *WDR62* gene (Fig. 2a). Findings from Whole Exome Sequencing (WES) were confirmed by Sanger sequencing. Reference sequence identifier for wild-type *WDR62* was obtained from GenBank; Accession: NG_028101, Version: NG_028101.1. Primers of exon 8 of *WDR62* (NM_001083961) were designed using Primer express software V3.0. The exon 8 of *WDR62* gene was amplified with Taq PCR Master Mix Kit (Qiagen Valencia, CA) using a standard amplification protocol, the sequencing reaction was set up with Big Dye Terminator 3.1 (Applied Biosystems) and remaining dye nucleotides were removed with SephadexTM G-50 superfine (GE Healthcare). Analysis of the amplicons was performed on automated sequencer Applied Biosystems Prism 3130 DNA Analyzer. Obtained sequences were aligned to the reference genome (GRCh37/hg19) using DNA Variant analysis software (Mutation Surveyor® software).

Segregation of the mutation in the family was confirmed by sequencing which showed, as expected, this mutation in the affected children and demonstrated that their parents carry this mutation in heterozygous state (Fig. 2b).

This mutation was previously unreported in various databases (dbSNP, 1000-genomes, Exome Variant Server) and also in our 40 samples of Moroccan exome data results (personal data). This mutation leads to a stop codon 343.

## Discussion and conclusions

Autosomal recessive primary microcephaly (MCPH) is a neurodevelopmental disorder characterized by a reduced head circumference present at birth and variable degrees of intellectual impairment. The current clinical definition of MCPH is as follows: (i): a head circumference of at least 4 SD below the age and sex-matched means; (ii): intellectual impairment that is not related to a neurological finding, such as spasticity or progressive cognitive decline; and (iii): most MCPH patients are of a normal height, weight, and appearance and have normal chromosome analysis and brain scan results [9–11].

The exact prevalence of MCPH is not known. The birth incidence of primary microcephaly ranges from 1.3 to 150/100000, depending on the population type and consanguineous populations [12].

Genetic heterogeneity underlies this disorder, 16 genes have been identified in MCPH; these include *ASPM* (MCPH1) [13], *WDR62* (MCPH2) [14] and others [15]. *ASPM* and *WDR62* are the two most common genes causative for MCPH [16].

We report in this study, two siblings suffering MCPH2 (MIM#604317). The clinical features of the two affected siblings were quite similar; they showed typical symptoms of MCPH such as microcephaly without brain malformations, and other neurological deficit such as considerable delayed fine motor skills, hyperactive behavior ans short stature. These clinical signs can expand the clinical description of MCPH. Through WES, we found a novel mutation in *WDR62* (c.1027C > T).

The *WDR62* gene (WD repeat domain 62) is localised inside the MCPH2 locus at chromosome 19q13.12 and contains 32 exons. *WDR62* encodes a 1523 amino acid protein with multiple WD40 domains at the N terminus. The protein WDR62 is expressed in neuronal precursor cells undergoing mitosis in the proliferative phase of neurogenesis and it has a role in cell proliferation, spindle organization, and mitotic progression, as well as in neuronal migration and in the regulation of neocorticogenesis [17, 18].

Furthermore, other authors discussed the WDR62 interaction partners that include Aurora and c-Jun N-terminal kinases as part of complex signalling mechanisms that may defines its neural functions [19].

Molecular evolution of *WDR62* has been studied and it showed that there is an acceleration of *WDR62* sequence evolution only in humans’ terminal branch relative to other mammals and also that amino acid substitutions are fixed in human population [20].

Mutations are distributed throughout the gene, and most of them result in complete loss of WDR62 protein. To date, 41 pathogenic mutations have been identified in *WDR62*, including eight nonsense mutations, four splicing mutations, 13 missense mutations, one intragenic deletion and 15 frameshift mutations (https://portal.biobase-international.com/hgmd/pro/gene.php?gene=WDR62).

Here, we report a novel homozygous mutation in the *WDR62* gene, which segregated as expected for a recessive pathogenic model. This mutation is a single base pair transition in exon 8 of the gene *WDR62* (c.1027C > T) resulting in a premature stop codon, causing early protein truncation.

Mutations in *WDR62* have been reported in a subset of patients with microcephaly, cortical malformations, and moderate to severe intellectual deficiency. Besides microcephaly, these patients had various brain malformations including callosal abnormalities, polymicrogyria, schizencephaly and subcortical nodular heterotopia [14, 16]; a subset has seizures [18]. Recently, Santasree Banerjee et al. 2016 reported a chineese girl with MCPH2 associated to dermatological phenotypes including early onset acanthosis and hyperkeratosis with two compound heterozygous mutations in *WDR62* [21].

The c.1027C > T (p.Gln343*) mutation in the *WDR62* gene was first reported to our best knowledge. Our study shows that WES is potentially reducing the time, effort, and cost associated with reaching a genetic diagnosis especially in a disorder with genetic heterogeneity such MCPH [22].

The results of our study will expand further the mutation spectrum of *WDR62* gene associated with MCPH. Our findings support WES as an effective diagnostic tool in families presenting with genetically heterogeneous disorders like MCPH. Mutational screening in different MCPH families from the Arab countries especially Morocco would help in genetic counselling and prenatal diagnosis for MCPH and would eventually enable us to reduce the incidence of MCPH in a highly consanguineous population.

## Abbreviations

MCPH: Autosomal recessive primary microcephaly
SD: Standard Deviations
WES: Whole Exome Sequencing

## References

[1] Kloepfer HW, Platou RV, Hansche WJ (1964). Manifestations of a recessive gene for microcephaly in a population isolate. J Genet Hum.

[2] Jaouad IC, Elalaoui SC, Sbiti A, Elkerh F, Belmahi L, Sefiani A (2009). Consanguineous marriages in Morocco and the consequence for the incidence of autosomal recessive disorders. J Biosoc Sci.

[3] Thornton GK, Woods CG (2009). Primary microcephaly: do all roads lead to Rome?. Trends Genet.

[4] Darvish H, Esmaeeli-Nieh S, Monajemi GB, Mohseni M, Ghasemi-Firouzabadi S, Abedini SS, Bahman I, Jamali P, Azimi S, Mojahedi F (2010). A clinical and molecular genetic study of 112 Iranian families with primary microcephaly. J Med Genet.

[5] Gul A, Hassan MJ, Hussain S, Raza SI, Chishti MS, Ahmad W (2006). A novel deletion mutation in CENPJ gene in a Pakistani family with autosomal recessive primary microcephaly. J Hum Genet.

[6] Gul A, Hassan MJ, Mahmood S, Chen W, Rahmani S, Naseer MI, Dellefave L, Muhammad N, Rafiq MA, Ansar M (2006). Genetic studies of autosomal recessive primary microcephaly in 33 Pakistani families: novel sequence variants in ASPM gene. Neurogenetics.

[7] Nicholas AK, Khurshid M, Desir J, Carvalho OP, Cox JJ, Thornton G, Kausar R, Ansar M, Ahmad W, Verloes A (2010). WDR62 is associated with the spindle pole and is mutated in human microcephaly. Nat Genet.

[8] Roberts E, Jackson AP, Carradice AC, Deeble VJ, Mannan J, Rashid Y, Jafri H, McHale DP, Markham AF, Lench NJ (1999). The second locus for autosomal recessive primary microcephaly (MCPH2) maps to chromosome 19q13.1-13.2. Eur J Hum Genet.

[9] Finlay BL, Darlington RB (1995). Linked regularities in the development and evolution of mammalian brains. Science.

[10] McCreary BD, Rossiter JP, Robertson DM (1996). Recessive (true) microcephaly: a case report with neuropathological observations. J Intellect Disabil Res.

[11] Mochida GH, Walsh CA (2001). Molecular genetics of human microcephaly. Curr Opin Neurol.

[12] Kaindl AM, Passemard S, Kumar P, Kraemer N, Issa L, Zwirner A, Gerard B, Verloes A, Mani S, Gressens P (2010). Many roads lead to primary autosomal recessive microcephaly. Prog Neurobiol.

[13] Bond J, Roberts E, Mochida GH, Hampshire DJ, Scott S, Askham JM, Springell K, Mahadevan M, Crow YJ, Markham AF (2002). ASPM is a major determinant of cerebral cortical size. Nat Genet.

[14] Bilguvar K, Ozturk AK, Louvi A, Kwan KY, Choi M, Tatli B, Yalnizoglu D, Tuysuz B, Caglayan AO, Gokben S (2010). Whole-exome sequencing identifies recessive WDR62 mutations in severe brain malformations. Nature.

[15] Rupp V, Rauf S, Naveed I, Windpassinger C, Mir A (2014). A novel single base pair duplication in WDR62 causes primary microcephaly. BMC Med Genet.

[16] Mahmood S, Ahmad W, Hassan MJ (2011). Autosomal recessive primary microcephaly (MCPH): clinical manifestations, genetic heterogeneity and mutation continuum. Orphanet J Rare Dis.

[17] Faheem M, Naseer MI, Rasool M, Chaudhary AG, Kumosani TA, Ilyas AM, Pushparaj P, Ahmed F, Algahtani HA, Al-Qahtani MH (2015). Molecular genetics of human primary microcephaly: an overview. BMC Med Genet.

[18] Yu TW, Mochida GH, Tischfield DJ, Sgaier SK, Flores-Sarnat L, Sergi CM, Topcu M, McDonald MT, Barry BJ, Felie JM (2010). Mutations in WDR62, encoding a centrosome-associated protein, cause microcephaly with simplified gyri and abnormal cortical architecture. Nat Genet.

[19] Shohayeb B, Lim NR, Ho U, Xu Z, Dottori M, Quinn L, Ng DCH (2017). The role of WD40-repeat protein 62 (MCPH2) in brain growth: diverse molecular and cellular mechanisms required for cortical development. Mol Neurobiol.

[20] Pervaiz N, Abbasi AA (2016). Molecular evolution of WDR62, a gene that regulates neocorticogenesis. Meta gene.

[21] Banerjee S, Chen H, Huang H, Wu J, Yang Z, Deng W, Chen D, Deng J, Su Y, Li Y (2016). Novel mutations c.28G>T (p.Ala10Ser) and c.189G>T (p.Glu63Asp) in WDR62 associated with early onset acanthosis and hyperkeratosis in a patient with autosomal recessive microcephaly type 2. Oncotarget.

[22] Rump P, Jazayeri O, van Dijk-Bos KK, Johansson LF, van Essen AJ, Verheij JB, Veenstra-Knol HE, Redeker EJ, Mannens MM, Swertz MA (2016). Whole-exome sequencing is a powerful approach for establishing the etiological diagnosis in patients with intellectual disability and microcephaly. BMC Med Genet.

